# A Gentle Hint to Non-Subscribing Correspondents

**Published:** 1850-04

**Authors:** 


### A GENTLE HINT TO NON-SUBSCRIBING CORRESPONDENTS.

The following bit of advice appears so appropriate, that we adopt it. We have our "occasional readers," too, and have sometimes to remind them of the true value of their patronage:—

"If 'An Occasional Reader' were to convert himself into 'A Constant Reader,' we feel confident that he would derive much benefit and consolation from the change. As it is our intention to comply with the hint of an 'Occasional Reader,' we hope that he will adopt our suggestion, and conclude his next communication with a more satisfactory signature."—*London Lancet.*

